# Digit ratios by computer-assisted analysis confirm lack of anatomical evidence of prenatal androgen exposure in clinical phenotypes of polycystic ovary syndrome

**DOI:** 10.1186/1477-7827-8-156

**Published:** 2010-12-29

**Authors:** Marla E Lujan, Amanda J Podolski, Donna R Chizen, Denis C Lehotay, Roger A Pierson

**Affiliations:** 1 Division of Nutritional Sciences, College of Agriculture of Life Sciences, Ithaca, NY 14853, USA; 2 Obstetrics, Gynecology & Reproductive Sciences, College of Medicine, University of Saskatchewan, Saskatoon, SK S7N0W8, Canada; 3 Pathology, College of Medicine, University of Saskatchewan, Saskatoon, SK S7N0W8, Canada

## Abstract

**Background:**

We recently showed that women with four clinical phenotypes of polycystic ovary syndrome (PCOS) do not demonstrate anatomical evidence of elevated prenatal androgen exposure as judged by a lower ratio of the index (2D) to ring (4D) finger. However, those findings conflicted with a previous study where women with PCOS had lower right hand 2D:4D compared to healthy female controls. Both these studies used Vernier calipers to measure finger lengths - a method recently shown to be less reliable at obtaining finger length measurements than computer-assisted analysis.

**Methods:**

Ninety-six women diagnosed with PCOS according to the 2003 Rotterdam criteria had their finger lengths measured with computer-assisted analysis. Participants were categorized into four recognized phenotypes of PCOS and their 2D:4D compared to healthy female controls (n = 48) and men (n = 50).

**Results:**

Digit ratios assessed by computer-assisted analysis in women with PCOS did not differ from female controls, but were significantly lower in men. When subjects were stratified by PCOS phenotype, 2D:4D did not differ among phenotypes or when compared to female controls.

**Conclusion:**

Computer-assisted measurements validated that digit ratios of women with PCOS do not show anatomical evidence of increased prenatal androgen exposure.

## Background

Polycystic ovary syndrome (PCOS) is a complex endocrine disorder, having no single diagnostic trait [1,2]. Much controversy has surrounded the diagnosis of this condition but in 2003 experts proposed that a diagnosis of PCOS be based on the presence of two of three symptoms: 1) oligo or chronic anovulation (amenorrhea), 2) biochemical and/or clinical hyperandrogenism and 3) polycystic ovaries on ultrasonography [1,2]. These criteria recognized the broad clinical spectrum of PCOS including, the manifestation four unique phenotypes [3]. Frank PCOS represents the most severe form of this condition and is characterized by the presence all three symptoms. Non-PCO PCOS is characterized by oligoanovulation, hyperandrogenism, but normal ovarian morphology. Ovulatory PCOS describes the presence of hyperandrogenism, polycystic ovaries and normal menstrual cycles, whereas Mild PCOS describes the presence of oligoamenorrhea and polycystic ovaries, but no hyperandrogenism. While the validity of these phenotypes is still being debated [4,5], there is consensus among experts that PCOS imparts serious consequences for the long-term health and quality of life of patients and therefore should invite early identification and intervention [1-6].

Despite familial clustering, the diverse manifestations of PCOS make it challenging to determine a single etiologic factor for this condition [7]. Experimental evidence in nonhuman primates has suggested that programming by prenatal androgens may contribute to variable susceptibility to PCOS in adult life and therefore lead to a heterogeneous clinical presentation (reviewed in [8]). Pregnant rhesus monkeys who received androgen treatment early in pregnancy (i.e. Day 40-44 of a 165-day pregnancy) gave arise to offspring that developed enlarged polycystic ovaries, hyperandrogenism, oligo-anovulation, increased basal luteinizing hormone (LH) secretion, insulin resistance, abdominal obesity, and hyperlipidemia. By contrast, the offspring of mothers that received treatment later pregnancy (i.e. after Day 90) did not demonstrate the same neuroendocrine alternations in LH secretion or changes in insulin sensitivity (reviewed in [8]). The differential effects of androgens during fetal development indicated that distinct programming windows existed for androgens to permanently modify future aspects of reproductive and metabolic function.

That prenatal androgens might contribute to development of PCOS has been retrospectively investigated by Cattrall et al. using a putative anatomical marker of in utero androgen exposure [9]. Exposure to androgens during fetal development affects finger length growth and leads to distinct discrepancies in male and female hand patterns (reviewed in [10]). Typically, men display a lower ratio between the index (2D) and ring (4D) fingers compared to women [11]. Cattrall et al. found a small, yet significant, difference in the right hand 2D:4D of women with PCOS compared to healthy female controls (98.3% of that in the controls) providing support for prenatal androgens in the etiology of PCOS [9]. More recently, we attempted to determine if clinical phenotypes of PCOS were associated with variations in 2D:4D but found no such difference [12]. The population studied by Cattrall et al. included only women with the most severe forms of PCOS, while our study encompassed a more varied patient population [9,12]. Yet, when the women participating in our study were stratified by clinical phenotype, we failed to detect a difference in 2D:4D, even in women with Frank PCOS [12].

To date, studies investigating 2D:4D in women with PCOS have used Vernier calipers to measure finger lengths. Recent studies have indicated that use of Vernier calipers is not the most reliable method of acquiring 2D:4D [13-15]. Vernier calipers demonstrate an interrater error of 1.2% or more [14], which has implications for comparing findings among studies [13]. Since the difference in right 2D:4D detected by Cattrall et al. may have fallen within the error range for Vernier calipers, we feel it important to reevaluate 2D:4D in women with PCOS using computer-based calipers which express an interrater error of <1% [14]. To that end, the aim of the current study was to re-evaluate the 2D:4D of our previous PCOS study population using the more reliable technique of computer-assisted analysis.

## Methods

### Study subjects

Women that had their finger lengths measured by Vernier calipers as part of a previous study [12] were invited to have their hands digitally scanned. Participants with PCOS had been recruited from women responding to an ad because of concerns over PCOS symptoms, as well as from those attending our Reproductive Endocrinology or Gynecology practice at the Royal University Hospital (Saskatoon, SK, Canada). Female control subjects had been recruited from women responding to an ad seeking healthy females of reproductive age with regular menstrual cycles. Of the 98 women diagnosed with PCOS that were invited to participate, 96 agreed to have their hands scanned at a follow-up visit. Of the 51 female controls subjects, 48 agreed to have their hands scanned.

All female participants had been clinically assessed for features of PCOS as previously described [12]. PCOS was defined by the 2003 Rotterdam criteria as having two of three symptoms: 1) oligo- or frank amenorrhea; 2) clinical and/or biochemical hyperandrogenism and 3) sonographic evidence of polycystic ovaries [1,2]. Oligo-amenorrhea was defined as an average cycle length of >35 days [16]. Hyperandrogenism was defined by a hirsutism score ≥8 using the modified Ferriman-Gallwey (FG) scale [17] and/or increased levels of serum androgens measured by mass spectroscopy (Free Androgen Index >10) [12]. Polycystic ovaries were defined as having a mean total follicle count of both ovaries >22.5 based on an internal ROC analysis which showed this value to have 90.7% sensitivity and 83.3% specificity to distinguish between normal and polycystic ovaries.

Fifty male participants were also recruited for this study. Males were primarily students and staff from University of Saskatchewan, Royal University Hospital and Cornell University. Males attested to being in good health but no clinical information was gathered from the male participants. Subjects with a history of injury or illness affecting their hands or fingers were not eligible to participate.

### Data collection

Participants were instructed to take off any jewelry or rings that would obstruct finger length measurements. The right and left hand of each participant was scanned in grey-scale using a Hewlett Packard scanner (Hewlett-Packard Company, Greeley, Colorado, USA). The volunteers gently positioned their hands on the surface of the scanner with fingers two through five placed parallel to one another and with their thumb directed outwards. Hands were scanned at 100 dpi to ensure that measurements were calibrated to a pixel per inch platform. A single observer analyzed the images using GIMP software (GNU Image Manipulation Program, version 2.6.4). The brightness/contrast of each image was subtly modified on an individual basis to help visualize the tips and proximal creases of the fingers as previously described [13]. The lengths of the index and ring fingers of each hand were then measured using mouse controlled calipers. In each instance, calipers were positioned midline along the finger's basal crease and expanded to the edge of the finger tip. Finger length measurements for all subjects were repeated one week later in order to assess intra-observer reliability. Digit ratios (2D:4D) for the left and right hands were computed by dividing the measurement of the index finger (2D) by that of the ring finger (4D). A two-way random intra-class correlation coefficient (ICC) analysis [18] was performed to assess intra-observer reliability using finger length measurements made on the same images on two separate occasions (i.e. one week apart). An ICC analysis showed 0.910, 0.921 and 0.947 degree of reliability in calculating left hand 2D:4D in women with PCOS, female controls and men. The degree of reliability in calculating right hand 2D:4D was 0.908, 0.910 and 0.928 in women with PCOS, female controls and men, respectively. Digit ratios for the left and right hands reported in this study denote the mean of the two measurements that were taken one week apart.

### Ethical considerations

This study was approved by the Biomedical Research Ethics Review Board at the University of Saskatchewan. All procedures conformed to the Canadian Tri-Council Guidelines for Human Research and International Good Clinical Practice Guidelines.

### Statistics

Descriptive statistics (mean ± standard deviation) of the 2D:4D for the left and right hands were tabulated for all women with PCOS, women with each clinical phenotype of PCOS, female controls, and men. Mean (±SD) clinical, hormonal and ultrasonographic characteristics of female subjects were also calculated. A two-way mixed ANOVA was performed to assess differences in 2D:4D and clinical features among groups. Effect sizes for 2D:4D data were described by Cohen's *d*. Linear regression analyses were used to examine associations between 2D:4D and clinical, hormonal and ultrasonographic features of PCOS. A p-value < 0.050 was regarded as statistically significant. Jmp 7 statistical software (SAS Institute Inc., Cary, NC, USA) was used to perform the statistical analysis.

## Results

### All PCOS vs. controls

Clinical features of all women meeting the criteria for PCOS and female controls are summarized in Table 1. Collectively, women with PCOS had higher BMI (p < 0.0001), elevated hirsutism (modified FG) scores (p < 0.0001), increased levels of total testosterone (p = 0.0048) and the free androgen index (p < 0.0001) compared to female controls. Total follicle counts (p < 0.0001) and ovarian volume (p < 0.0001) were increased in women with PCOS compared to female controls and they also reported longer intervals between menstrual cycles (p < 0.0001) compare to female controls.

**Table 1:** A comparison of clinical features among women with PCOS and female controls.

	PCOS (n = 96)	Controls (n = 48)	P-value
Age (y)	28.3 ± 4.5	26.9 ± 4.2	0.0684
BMI (kg/m^2^)	32.2 ± 8.4	24.1 ± 3.7	<0.0001
Modified FG Score*	10.7 ± 6.6	3.3 ± 2.5	<0.0001
Total Testosterone (nmol/l)	3.6 ± 1.8	2.8 ± 0.9	0.0048
Free Androgen Index (%)	12.4 ± 9.4	5.7 ± 2.9	<0.0001
Total Follicle Count	41.1 ± 15.0	15.7 ± 5.5	<0.0001
Ovarian Volume (ml)	9.7 ± 3.2	6.1 ± 2.3	<0.0001
Menstrual Cycle Length (d)	109.0 ± 99.7	29.7 ± 3.0	<0.0001

* Modified Ferriman-Gallwey (FG) scale used to denote level of hirsutism.

**Figure 1:**
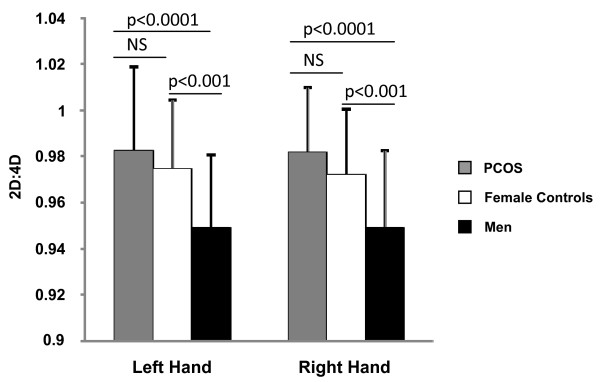
**Digit ratios of women with PCOS, female controls, and men**. Digit ratios did not differ between women with PCOS and female controls (NS; not significant). Digit ratios in men were lower than women with PCOS (p < 0.0001) and female controls (p < 0.001). All data points represent Mean ± SD.

Figure 1 compares mean 2D:4D of left and right hands of all women with PCOS and both female and male controls. Left 2D:4D were 0.982 ± 0.030, 0.974 ± 0.037, and 0.949 ± 0.032, and right 2D:4D were 0.981 ± 0.028, 0.972 ± 0.028, and 0.949 ± 0.034, respectively. Left (p = 0.363) and right (p = 0.146) 2D:4D did not differ between women with PCOS and female controls. By contrast, left and right 2D:4D for men were significantly lower than 2D:4D in women with PCOS (Left Hand, *d *= -1.076; Right Hand, *d *= -1.252) and female controls (Left Hand, *d *= -0.741; Right Hand, *d *= -0.918).

### PCOS phenotypes vs. controls

Of the 96 women with PCOS, 63 were classified as having Frank PCOS (66%), 4 as Non-PCO PCOS (4%), 19 as Ovulatory PCOS (20%) and 10 as Mild PCOS (10%). Table 2 corroborates the designation of these phenotypes by presenting the corresponding clinical, hormonal and ultrasonographic data for participants in each of the four phenotypes. Women with Frank PCOS had higher hirsutism (modified FG) scores (p < 0.0001), increased levels of testosterone (p = 0.022) and the free androgen index (p < 0.0001), larger follicle counts (p < 0.0001) and ovarian volume measurements (p < 0.0001) and longer menstrual cycle lengths (p < 0.0001) compared to female controls. Women with Non-PCO PCOS had higher indices of hyperandrogenism (FG score, p = 0.013) and longer menstrual cycles (p = 0.050) compared to female controls but were similar in follicle counts (p = 1.00) and ovarian volume (p = 0.955) measurements compared to controls. Women with Ovulatory PCOS reported menstrual cycle lengths similar to female controls (p = 1.00) but their hirsutism scores (p < 0.0001), total follicle counts (p < 0.0001) and ovarian volumes (p = 0.003) were higher than controls. Lastly, women with Mild PCOS had longer menstrual cycles (p = 0.014), higher follicle counts (p < 0.0001) and increased ovarian volumes (p = 0.031) compared to female controls but were similar in their indices of hyperandrogenism compared to controls.

**Table 2:** A comparison of clinical features and mean digit ratios (2D:4D) among women with four clinical phenotypes of PCOS and female controls.

	All PCOS (n = 96)	Frank (n = 63)	Non-PCO (n = 4)	Ovulatory (n = 19)	Mild (n = 10)	Controls (n = 48)
Age (y)	28.3 ± 4.5	27.8 ± 4.5^a^	31.4 ± 4.4^a^	29.3 ± 4.7^a^	28.5 ± 3.9^a^	26.9 ± 4.2^a^
BMI (kg/m^2^)	32.2 ± 8.4	33.5 ± 8.2^a^	40.1 ± 5.3^a^	31.4 ± 7.1^a^	22.2 ± 4.6^b^	24.1 ± 3.7^b^
Modified FG Score*	10.7 ± 6.6	11.9 ± 7.0^a^	12.0 ± 3.9^a^	10.7 ± 3.8^a^	2.3 ± 2.7^b^	3.7 ± 2.9^b^
Total Testosterone (nmol/l)	3.6 ± 1.8	3.8 ± 2.0^a^	2.9 ± 1.7^a, b^	2.7 ± 0.6^a, b^	3.3 ± 1.2^a, b^	2.8 ± 0.9^b^
Free Androgen Index (%)	12.4 ± 9.4	15.3 ± 10.1^a^	9.9 ± 3.7^a, b^	6.8 ± 3.1^b^	4.3 ± 1.8^b^	5.7 ± 2.9^b^
Total Follicle Count	41.1 ± 15.0	44.0 ± 15.1^a^	15.6 ± 3.1^c^	35.9 ± 10.4^b^	44.3 ± 12.1^a, b^	15.7 ± 5.5^c^
Ovarian Volume (ml)	9.7 ± 3.2	10.1 ± 3.4^a^	7.2 ± 3.0^a, b^	9.1 ± 2.9^a^	9.1 ± 2.1^a^	6.1 ± 2.3^b^
Menstrual Cycle Length (d)	109.0 ± 99.7	127.4 ± 98.3^a^	75.5 ± 12.5^a^	31.7 ± 4.7^b^	101.1 ± 117.2^a^	29.7 ± 3.0^b^
Left Hand 2D:4D	0.982 ± 0.030	0.982 ± 0.029^a^	0.981 ± 0.060^a^	0.982 ± 0.028^a^	0.983 ± 0.032^a^	0.974 ± 0.037^a^
Right Hand 2D:4D	0.981 ± 0.028	0.981 ± 0.029^a^	0.965 ± 0.028^a^	0.986 ± 0.027^a^	0.983 ± 0.027^a^	0.972 ± 0.028^a^

Note: A column representing data for all women with PCOS has been added for ease of comparison. Within each row, assignment of the same letter represents no significant difference between groups; *The modified Ferriman-Gallwey (FG) scale was used to denote level of hirsutism.

**Figure 2:**
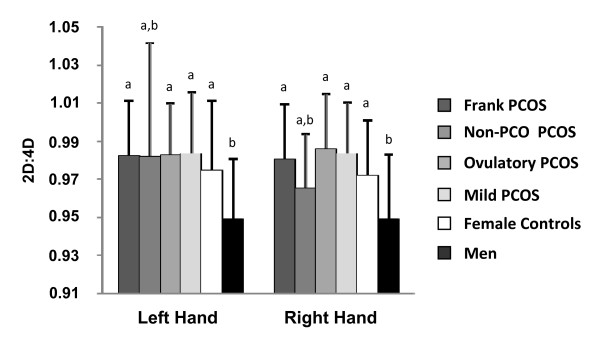
**Digit ratios of women with four clinical phenotypes of PCOS, female controls, and men**. Digit ratios did not differ between the various phenotypes of PCOS and female controls. Digit ratios in men were lower than PCOS groups (p < 0.03) and female controls (p < 0.01). All data points represent Mean ± SD. Assignment of the same letter represents no significant difference between groups.

Mean left and right 2D:4D for each clinical phenotype and the female controls are summarized in Table 2 and presented in Figure 2. Left and right 2D:4D were similar among clinical phenotypes of PCOS and female controls (Comparisons for all pairs, p > 0.450). By contrast, male 2D:4D were significantly lower than 2D:4D in women with Frank (Left Hand, *d *= 1.090, p < 0.0001; Right Hand, *d *= -1.042, p < 0.0001), Ovulatory (Left Hand, *d *= -1.118, p = 0.003; Right Hand, *d *= -1.259, p < 0.0001) and Mild PCOS (Left Hand, *d *= -1.074, p = 0.031, Right Hand, *d *= -1.156, p = 0.007) and female controls (Left Hand, *d *= -0.741, p = 0.031, Right Hand, *d *= -0.918, p = 0.003).

### Digit ratios and clinical features of PCOS

Associations between 2D:4D and signs and symptoms of PCOS are summarized in Table 3. Digit ratios of the left and right hands of female participants were not associated with variations in BMI, length of the menstrual cycle, total follicle counts or ovarian volume measurements. By contrast, weak positive correlations were detected between 2D:4D and measures of hyperandrogenism including hirsutism scores (Left Hand, p = 0.017), total testosterone (Left Hand, p = 0.005; Right Hand, p = 0.023) and the free androgen index (Left Hand, p = 0.005; Right Hand, p = 0.021).

**Table 3:** Associations between digit ratios (2D:4D) and clinical features of PCOS.

	R _Left 2D:4D_	P-value	R _Right 2D:4D_	P-value
BMI (kg/m^2^)	0.144	0.092	0.102	0.231
Modified FG Score*	0.203	0.017	0.158	0.062
Total Testosterone (nmol/l)	0.251	0.005	0.204	0.023
Free Androgen Index (%)	0.256	0.005	0.208	0.021
Total Follicle Count	0.087	0.301	0.093	0.275
Ovarian Volume (ml)	0.097	0.255	0.097	0.253
Menstrual Cycle Length (d)	0.033	0.703	-0.013	0.881

*Modified Ferriman-Gallwey (FG) scale used to denote level of hirsutism.

## Discussion

The goal of this study was to address the controversy over evidence of prenatal androgen exposure reflected in the digit ratios of women with PCOS. Recently our group showed that when 2D:4D were measured with Vernier calipers, women with PCOS did not demonstrate finger length patterns consistent with increased levels of in utero androgen exposure [12]. This was in contrast to a previous report that had also used Vernier calipers to measure 2D:4D in women with PCOS [9]. Since observed differences in 2D:4D are generally small, there is growing support that studies investigating potential effects of prenatal androgens use the most consistent and reliable technique available to measure finger lengths [13-15,18-20]. In this study, we imaged the hands of women with four clinical phenotypes of PCOS, healthy female controls and men, and used computer-based calipers to measure their finger lengths since this method was recently validated to be the most reliable [14,15]. Consistent with this being the most reliable technique, we obtained levels of intra-observer reliability when calculating 2D:4D (Mean ICC = 0.912) that were much higher than what we obtained when we used Vernier calipers to make measurements in this same study population (Mean ICC = 0.845) [12]. These levels of reliability were in range of what others have demonstrated in studies comparing the appropriateness of computer-assisted measurements for obtaining 2D:4D in women [14].

In the current study, a comparison of 2D:4D among healthy female controls and women diagnosed with PCOS by the Rotterdam criteria showed no significant difference among groups. Unlike a previous report of lower right-hand 2D:4D in women with PCOS [9], we again noted a tendency toward lower 2D:4D in healthy female controls which would contradict a theory supporting excess prenatal androgen exposure in women with clinically defined PCOS. A tendency for lower 2D:4D in female controls contributed to our paradoxical finding that 2D:4D were positively, albeit weakly, associated with clinical markers of androgen excess in women. Our female control subjects were carefully selected following a complete clinical assessment for PCOS and they showed no indications of androgen excess, menstrual cycle irregularity or ovarian dysmorphology. The inclusion of men to our study population afforded us the possibility to compare 2D:4D between men and women with or without PCOS. This comparison substantiated that our female control group had 2D:4D that were consistent with what one would expect when comparing 2D:4D among sexes [11]. Moreover, our finding of lower 2D:4D in men compared to women with PCOS further supported the notion that women with PCOS do not demonstrate androgenized finger lengths.

Since the PCOS criteria we used spanned a broad clinical spectrum - with a potentially variable etiology - we stratified subjects by phenotype and compared 2D:4D among PCOS groups. Our hypothesis that variable manifestations of PCOS might reflect variations in 2D:4D was not supported even when using this more reliable technique to obtain finger length measurements. That is, even women with severe forms of the syndrome had finger length patterns that were no different than milder variants of PCOS or female controls. It is important to acknowledge that the interpretation of these finding is limited by the small number of subjects available for analysis in each of the phenotypic groups. Comparisons of 2D:4D among all women with PCOS and controls were sufficiently powered in that they had greater than 80% power to detect a difference of 0.019 in 2D:4D at an alpha level of 0.05 assuming a standard deviation of 0.035 [9,12]. By contrast, comparisons of 2D:4D in women with Frank, Non-PCO, Mild or Ovulatory PCOS all had less than 22% power to detect differences among groups. Assuming the afore mentioned effect size and standard deviation, a minimum of 55 subjects per group would be required to perform an adequately powered assessment of digit ratios among PCOS phenotypes. There were several obstacles that limited our ability to recruit the desired number of subjects. First, we were limited in the number of women with PCOS that could be identified in a small geographical area. The prevalence of PCOS is estimated at 5 - 10% [3] and while that may appear to represent a relatively common condition, our ability to locate women that were sufficiently concerned over PCOS symptoms and willing to participate in a clinical study was limited. Second, the identification of PCOS phenotypes is an incredibly involved process requiring participation from various clinical specialists. Difficulty in coordinating access to resources limited the number of subjects we could assess at any given time. Last, it may not have been even possible to identify the necessary number of subjects meeting the criteria for each PCOS phenotype. This was likely the case for the Non-PCO PCOS group. Diagnostic criteria for PCOS are controversial as are the manifestation of these individual phenotypes [4,5]. Over a two-year period, we screened over 125 women with concerns of PCOS symptoms and only identified 4 women meeting the criteria for Non-PCO PCOS. At this rate, it would have taken us over 25 years to identify the appropriate number of subjects. The low prevalence of this phenotype calls into question its validity and the need to verify ultrasonographic criteria for polycystic ovarian morphology [21-23].

There is growing concern that 2D:4D may represent only a weak, or perhaps even useless, measure of prenatal androgen exposure [24-27]. It is difficult to know for certain whether our negative findings represent a lack of involvement of prenatal androgens in PCOS or whether this represents a lack of an effect of androgen exposure on 2D:4D per se. That prenatal androgens directly contribute to the development of PCOS in humans seems increasingly unlikely. Experimental evidence showing that pregnant monkeys receiving exogenous androgens gave rise to female offspring with PCOS symptoms is the best data supporting a role for prenatal androgens in PCOS [8]. However, very little is known about the mechanism(s) whereby prenatal androgen excess leads to PCOS-like alterations in reproductive and metabolic function. In humans, it is unlikely that maternal androgens sufficiently cross the placental barrier to affect the fetus unless placental function is compromised in some capacity in PCOS mothers [8]. Another possibility is a fetal origin of androgen excess in which increased androgen production from the fetal adrenals and/or ovaries act during development to program future organ systems [8]. There is no evidence to support either of these possibilities and the most recent study of opposite-sex twin pairs did not support an increased risk of PCOS in the female twin despite exposure to intrauterine androgens of the male fetus [28]. Given that our study was limited by small sample sizes, it will be important to revisit this issue with an adequately powered study using non-controversial criteria for PCOS - if and when they come to fruition.

## Conclusions

Computer-based measurements of 2D:4D verified that women with clinical phenotypes of PCOS do not exhibit anatomical indications of increased prenatal androgen exposure. The current study also extends our previous observations to show that 2D:4D in women with PCOS were higher than those in men, consistent with PCOS patients having relatively lower levels of in utero androgen exposure. We caution that these results do not entirely rule out the possibility that in utero androgens play a role in the development of PCOS; however, androgenized 2D:4D are not a characteristic of PCOS.

## Competing interests

The authors declare that they have no competing interests.

## Authors' contributions

MEL conceived, designed and coordinated the study, and drafted the final manuscript. AJP coordinated the study, conducted the statistical analyses and drafted the final manuscript. DRC clinically evaluated the study volunteers. DCL developed and performed the endocrine assays. RAP participated in the conception and design of the study and provided resources and equipment to complete the study. All authors read and approved the final manuscript.
